# Impact of Maternal Lifetime Stress on Offspring Biological Aging: A Systematic Review and Meta-Analysis of Observational Studies

**DOI:** 10.3390/ijms27073019

**Published:** 2026-03-26

**Authors:** María Loreto Muñoz Venegas, Miriam Shasa Quiccione, Sukshma Sharma, Francesco Gianfagna, Francesca Bracone, Paola De Domenico, Alfonsina Tirozzi, Chiara Cerletti, Maria Benedetta Donati, Giovanni de Gaetano, Licia Iacoviello, Alessandro Gialluisi

**Affiliations:** 1 Research Unit of Epidemiology and Prevention, IRCCS Neuromed, Via dell’Elettronica, 86077 Pozzilli, Italy; lorettomunozepi@gmail.com (M.L.M.V.); alessandro.gialluisi@gmail.com (A.G.); 2 EPIMED Research Center, Department of Medicine and Surgery, University of Insubria, 21100 Varese, Italy; 3 Medical Directorate, Istituti Clinici Scientifici Maugeri IRCCS, 21049 Tradate, Italy; 4 Istituto Clinico Mediterraneo, 84043 Agropoli, Italy; 5 Medical Genetics Unit, Department of General and Emergency Pediatrics, AORN Santobono-Pausilipon, 80122 Naples, Italy; 6 Department of Medicine and Surgery, LUM University, 70010 Casamassima, Italy

**Keywords:** telomere length, DNA methylation age, maternal stress, depression, anxiety, trauma, offspring, pregnancy, in utero exposome

## Abstract

Maternal stress during lifetime and pregnancy may influence offspring epigenetic age, impacting long-term health. We conducted a systematic review and meta-analysis of associations between maternal stress and epigenetic aging markers: telomere length (TL) and DNA methylation (DNAm) age acceleration. The systematic search was performed according to PRISMA guidelines and registered on PROSPERO (ref. CRD42023474640). Fixed and random effect meta-analyses were carried out, stratified by stress type and marker type (TL, DNAm). Sixteen studies met inclusion criteria; 12 were meta-analyzed (10 TL, 2 DNAm). Due to high heterogeneity, restricted maximum likelihood meta-analysis suggested significant inverse associations between maternal stress and offspring TL. Perceived stress was associated with shorter TL (*p*-value = 7 × 10^−4^, β = −0.085, 95%CI [−0.135, −0.036]), as was lifetime stress/trauma (*p*-value = 0.01, β = −0.209, 95%CI [−0.370, −0.049]). In contrast, maternal stress showed no significant associations with DNAm age acceleration (*p*-value = 0.32). Both perceived maternal stress and maternal stress were associated with shorter offspring TL, suggesting that stress exposure across the maternal lifespan influences offspring biological aging markers. No significant association was observed with DNAm-based aging clocks. Further studies with larger sample sizes and more homogeneous settings are needed to confirm and expand upon our observations.

## 1. Introduction

The developmental origins of health and disease (DOHaD) hypothesis postulates that the in utero environment can predispose individuals to develop certain diseases later in life, including neurodevelopmental disorders [1,2], metabolic syndrome, fatty liver, and depressive disorders [3,4]. Among the various maternal exposures during pregnancy, psychological stress has emerged as a potential factor that can disrupt fetal programming [5]. Maternal exposures such as prenatal stress, psychosocial stress, and xenobiotics can cause an increase in pro-inflammatory cytokines and abnormal epigenetic modifications during pregnancy [3] which can ultimately impact offspring health.

While the classical DOHaD framework focuses on the prenatal period as a critical window of development, a more comprehensive, life-course perspective is needed. A mother’s physiological and psychological state during pregnancy is not an isolated event but is shaped by her cumulative stress and adversity across her own lifespan, from childhood through adulthood [6]. Furthermore, early-life stress can calibrate the responsivity of the hypothalamic–pituitary–adrenal (HPA) axis [7], while stress in adulthood contributes to allostatic load [8]. Both could influence the in-utero environment and potentially the germline, thereby affecting fetal development through both direct programming and intergenerational pathways.

Therefore, restricting the focus solely to pregnancy may overlook foundational influences on maternal biology that could ultimately influence an offspring’s developmental trajectory [9]. To fully understand the developmental origins of accelerated biological aging, it is necessary to expand the scope and encompass maternal lifetime stress [8,10].

One potential consequence of a suboptimal in utero environment is the acceleration of biological aging. Biological aging is defined as the discrepancy between the chronological age (CA) and biological age (BA), and this difference has been reported to predict better aging-related clinical outcomes, including mortality, hospitalization risk, and well-being [11]. Among the non-genetic factors that influence biological aging, mental health and stress have been hypothesized to accelerate the biological aging rate [12,13,14]. So far, studies have used the two most investigated biological aging molecular markers, namely: (1) telomere length (TL) [15], and (2) DNA methylation-based epigenetic clocks [14].

Notably, telomeres are the protective caps found at the ends of genomic DNA, which shorten at each cell division and, therefore, reflect the age of a cell. In particular, leukocyte TLs are used because their age-associated shortening is commonly regarded as an important contributor to organismal aging [15,16,17] in addition to being readily available and convenient to handle [18]. DNA methylation (DNAm) age acceleration, commonly measured through epigenetic clocks, predict biological age based on DNAm changes (hypo-/hypermethylation) accumulating in specific sites of the genome [17]. Since DNAm involves the addition of a methyl group to the five-prime (5′) position on cytosines in cytosine-guanine dinucleotide sites (CpGs), these clocks predict age or age-related phenotypes by combining the methylation values of tens to hundreds of CpGs selected using different statistical approaches [13,17].

These biomarkers are hypothesized to be sensitive to the physiological processes dysregulated by maternal stress [5]. Specifically, persistent prenatal maternal adversity is documented to disrupt the hypothalamic–pituitary–adrenal (HPA) axis, culminating in increased circulating glucocorticoids [19]. These stress hormones can cross the placenta and may directly impact the fetus, potentially inhibiting telomerase activity, accelerating telomere attrition, and altering the establishment of epigenetic patterns, thereby leaving a molecular signature of accelerated aging at birth [15,20,21]. Despite these promising findings, evidence concerning maternal prenatal stress remains unclear [22,23]. For instance, the term “maternal prenatal stress” is broadly defined as any stressful life exposure of the expectant mother [24], including personal life, job status, housing, or domestic violence [19]. More so, previous literature has suggested that maternal prenatal stress profoundly affects offspring [13,15,17,19,22,24] by influencing fetal health, possibly through cortisol level regulation, lifestyle changes, and epigenetic patterns [25,26,27]. Moreover, the literature has found conflicting evidence regarding the association between mothers’ lifetime (childhood, adolescence, adulthood, and pregnancy) stress and the offspring’s TL and DNAm-based epigenetic clocks [28,29,30].

Findings for DNAm age acceleration are heterogenous with studies reporting acceleration in some contexts [31] and deceleration in others [32,33]. This inconsistency could be due to methodological variations across studies, including the use of different stress instruments and constructs (e.g., perceived stress, depression, lifetime trauma), the timing of stress assessment (pregnancy-specific versus lifetime), or the child’s age at outcome measurement [28,29,30].

Therefore, to clarify this complex relationship and quantify the overall evidence, our study aimed to examine the associations between maternal stress and the two most common molecular measures of biological aging in the offspring, namely TL and DNAm age acceleration. This systematic review and meta-analysis of 16 studies (12 meta-analyzed) revealed that maternal stress during pregnancy is associated with shorter offspring TL, a biomarker of accelerated biological ageing. Notably, we identified a significant inverse association between reported maternal stress and TL, irrespective of the timing of exposure to stressors in the mothers’ lives. While evidence for DNAm age acceleration remains limited by the small number of available studies (*n* = 2), these findings suggest potential intergenerational transmission of a stress-related biological ageing signature.

While we primarily used the term “maternal stress” defined as the physiological and behavioral response that occurs when acute or chronic psychosocial difficulties overwhelm the coping abilities that allow a woman to return to physiological homeostasis [34], we acknowledge that this umbrella term can extend to various forms of psychological adversity, including perceived stress, depressive symptoms, anxiety, post-traumatic stress disorder (PTSD), and lifetime trauma. In this context, “maternal stress” would broadly align with the concept of “maternal psychosocial adversity,” a term used in the DOHaD field that encompasses both psychological and social dimensions, reflecting the multidimensional nature of the exposures investigated herein [35].

## 2. Methods

### 2.1. Protocol Registration

This systematic review was performed according to the PRISMA guidelines [36] and was registered on PROSPERO (reference CRD42023474640) (available online: https://www.crd.york.ac.uk/prospero/display_record.php?ID=CRD42023474640, accessed on 1 March 2026).

### 2.2. Information Sources and Search Strategy

A systematic literature search was conducted on 30 November 2024, across four electronic databases namely EMBASE, PubMed, MEDLINE (both via Ovid), and Scopus. The search strategy was designed and executed in accordance with PRISMA guidelines. To ensure comprehensiveness, our strategy incorporated a combination of controlled vocabulary (e.g., Medical Subject Headings (MeSH) terms in PubMed/MEDLINE, EMTREE terms in EMBASE) and free-text keywords related to three core concepts: maternal exposure: e.g., “Mothers”, “Pregnant Women”, maternal, prenatal; psychological stress: e.g., “Stress, Psychological”, depression, anxiety, adversity, trauma and; offspring epigenetic aging: e.g., “Telomere”, “DNA methylation”, “epigenetic clock,” “biological age”, “telomere length”. These concepts were combined using the Boolean “AND” operator. No date or language restrictions were applied to maximize sensitivity, and the search was limited to human studies. The full, reproducible search strategy for each database, including all specific terms and field codes, is shown in Supplementary Table S1. This final strategy was informed and validated by iterative, exploratory searches carried out throughout the study’s development phase.

The full-text versions were stored as PDF files, and all the studies extracted were managed and stored using Rayyan Software [37] a web-based systematic review tool, and Reference Manager Zotero v7.0.15 (https://www.zotero.org/).

#### 2.2.1. Inclusion Criteria

We included observational studies, including cohort studies and cross-sectional studies examining the association between lifetime (childhood, adolescence, adulthood, and pregnancy) stress of mothers and offspring’s biological age assessed by TL or DNAm. The current systematic review included only studies that reported results for women ≥ 16 years of age. Other inclusion criteria included availability of offspring’s TL and/or DNAm age measures and availability of stress assessment through validated psychometric scales: Edinburgh Postnatal Depression Scale (EPDS), Spielberger State-Trait Anxiety Inventory (STAI), Holmes and Rahe Stress Scale questionnaire, 10-item Pregnancy-specific Stress Scale (PSS-10), 80-item Crisis in Family Systems–Revised (CRISYS-R) survey, Adverse Childhood Experience questionnaire score (ACE), Trauma History Questionnaire, Life Events Scale (LES), World Mental Health Life Events Questionnaire (LEQ), the Childhood Trauma Questionnaire (CTQ) and, the Intimate Partner Violence (IPV) Questionnaire. Psychological distress was assessed using the Self-Reporting Questionnaire (SRQ-20), Structured Trauma-Related Experiences and Symptoms Screener for Adults (STRESS-A), and the Difficulties in Emotion Regulation Scale (DERS).

#### 2.2.2. Exclusion Criteria

We excluded studies for the following: (1) abstracts, systematic review articles, umbrella, scoping and literature reviews, editorials, case reports, letters, surveys, conference papers, theses, randomized controlled trials, and pre-clinical studies, and (2) studies including women reporting a diagnosis or history of psychiatric disorders, or any substance dependence other than nicotine during pregnancy or otherwise.

### 2.3. Definition of Outcomes

In this systematic review and meta-analysis, we considered two main measures of biological aging in offspring as outcomes: TL (from cord blood leukocytes, saliva, or placenta) measured as T/S ratio (estimated by comparing the amount of DNA of telomere repeat amplification product (T) to a single copy gene (S) product such as beta-globin or albumin) [38], and DNAm aging of the offspring which is defined as the discrepancy between DNAm and chronological age of a subject based on DNA extracted from cord blood leukocytes, saliva, or venous blood [39,40,41]. TL was measured using various techniques across the included studies, including quantitative polymerase chain reaction (qPCR) and Telomere Restriction Fragment (TRF), and flow (see Supplementary Table S6 for a comparison).

### 2.4. Selection Process

**Figure 1:**
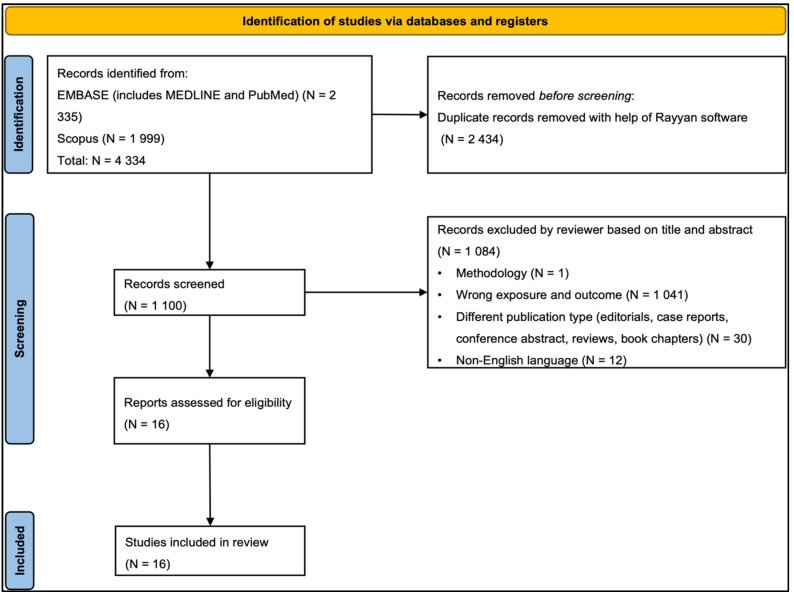
PRISMA flowchart. Abbreviations: N: sample size.

The studies were selected according to title and abstract using the Rayyan Software tool [37], which was also used to identify duplicate articles from all databases. Two independent reviewers (MSQ and SS), using the Rayyan software, performed an initial screening according to the title and abstract. Then, the two reviewers (MSQ and SS) performed further screening to reduce selection bias and retrieved the full-text versions of the studies selected according to the inclusion and exclusion criteria (see PRISMA flowchart, Figure 1). A third independent reviewer (AG) was consulted in case of any disagreement.

### 2.5. Data Extraction and Data Items

The following data were extracted from the included studies: (a) study characteristics (Table 1) with inclusion and exclusion criteria and participant details (Table 2); and (b) exposure measures and timing (Table 3) with outcomes and associations found (Table 4).

### 2.6. Quality Assessment Process

We used the Joanna Briggs Institute (JBI) Critical Appraisal Checklist for Analytical Cross-Sectional Studies [54] to assess the risk of bias in included studies. Two separate JBI checklists for cohort studies (11 questions) and cross-sectional studies (8 questions) were used. The JBI checklist included questions addressing the internal validity and risk of bias of study designs, particularly confounder selection, and use of appropriate statistical analysis. The quality of the included studies was assessed by three independent reviewers (MSQ, SS, and AG) who had to select “Yes,” “No,” “Unclear,” or “Not Applicable” in the JBI tool. Any disagreements were mutually resolved by analyzing the details of the article.

### 2.7. Meta-Analysis

Building upon the systematic review previously outlined, a meta-analysis was computed to quantitatively incorporate our findings into the association between maternal stress and offspring epigenetic outcomes, specifically focusing on TL and DNAm age acceleration. While our registered PROSPERO protocol (CRD42023474640) indicated a primary focused on the Perceived Stress Scale (PSS), our systematic literature search revealed that limiting to PSS would exclude substantial relevant evidence on maternal stress and offspring epigenetic aging. We therefore expanded our inclusion criteria to encompass a broader range of validated maternal psychological stress measures. This refinement maintains our conceptual coherence, while allowing comprehensive assessment of maternal psychological adversity across different measurement approaches and temporal windows. The PROSPERO protocol has been amended to reflect these expanded criteria. This decision, made prior to data extraction and analysis aligns with PRISMA guidelines, which allows for methodological refinement. Consequently, for our primary meta-analysis, we grouped these related exposures, including perceived stress, depressive symptoms, anxiety, and lifetime trauma, under the broader umbrella term “maternal stress” to ensure sufficient statistical power. We also conducted exploratory, stress-type-specific meta-analysis where the number of studies permitted (k ≥ 3).

Statistical analyses were carried out by an independent author (MLMV) to avoid any potential bias derived by previous knowledge of the literature. After our systematic literature review search, we extracted and curated study-level data using R statistical programming (version 4.4.3) and the RStudio integrated development environment (version 2024.12.1+563). All input values for the meta-analysis were directly based on the numbers presented in the published articles. The tidyr [55], metafor [56], metap [57], ggplot2 [58] and dplyr [59] R-packages were used for our calculations and analysis. The extracted variables used included reported effect sizes (expressed as beta (β) coefficients), standard errors (SE), 95% confidence intervals (CI), *p*-values, and sample sizes for each included study.

To ensure data integrity and comparability across studies, we applied a rigorous data cleaning and quality control protocol. This protocol involved solving formatting inconsistencies (e.g., Unicode minus signs, inconsistent SE notation, etc.), removing any non-numeric entries, and, where possible, estimating missing SE values based on reported CI using the formula $SE=\frac{\left(Upper\;CI\;Boundary\;-\;Lower\;CI\;Boundary\right)}{2\;\times \;1.96}$. Subsequently, the studies were categorized into distinct analytical subgroups based on specific type of maternal stressors (i.e., perceived stress, depression, symptoms of depression, history of lifetime trauma, PTSD) and the measured biological outcome (TL versus DNAm aging biomarkers). These group-specific analyses were pre-defined based on our systematic literature search results. In cases of overlapping data from the same cohort, we selected the estimate from the analysis with the larger sample size and/or more comprehensive adjustment for confounders.

A key methodological consideration was the conceptual heterogeneity of maternal stress exposures reported in the literature, which encompassed distinct constructs such as perceived stress, depressive symptoms, anxiety, PTSD, and lifetime trauma. As mentioned above, in our primary analysis, we strategically grouped these exposures under the umbrella term “maternal stress” to ensure sufficient statistical power for a quantitative synthesis. However, we conducted additional exploratory meta-analyses stratified by these specific stressor types to investigate whether the associations differed across distinct psychological constructs.

Meta-analyses were then performed using both fixed-effects and random-effects models, implemented via the rma() function within the metafor [56] R-package. Heterogeneity across studies involved the calculation of heterogeneity (I^2^) statistic, between-study variance (τ^2^), and the computation of Cochran’s Q statistic along with its associated *p*-values. To complement model-based approaches, we also applied *p*-value combination methods, namely Fisher’s and Stouffer’s methods. These were calculated through both manual computation and using the metap [57] R-package (using the sumlog and sumz functions). Where appropriate, sample-size weighting was also used in fixed-effects models. For groups with fewer than three independent studies, formal model-based effect size estimates were omitted; instead, only the results of the *p*-value combination methods were reported. Subgroup analyses were performed separately for TL and DNA methylation age acceleration outcomes to further explore potential differential effects. For DNAm age clocks, an exception was made to the k ≥ 3 threshold for exploratory purposes, given the scarcity of available studies and the novelty of the analysis. Associations were considered significant if they survived correction for multiple testing of two different offspring biological aging outcomes (α = 0.025) and were stable across fixed and random-effect meta-analysis approaches.

To assess the potential for small-study effects and publication bias, we used funnel plots for meta-analyses that had enough studies. For the association between “maternal perceived stress and offspring TL” (ten studies), a visual inspection of the funnel plot did not reveal substantial asymmetry. In contrast, this diagnostic was not performed for the association between “maternal stress and DNAm age acceleration” due to an insufficient number of studies (two studies). Further, formal publication bias assessment was conducted using Begg’s rank correlation [60,61] and Egger’s regression test [62] for groups with sufficient studies (k ≥ 3).

To explore potential sources of heterogeneity observed in the main meta-analysis, we performed a meta-regression analysis. Given the recommendation in the Cochrane Handbook [63] which suggests using a minimum of ten studies as desirable for each study-level variable, our analysis was limited. We had five studies within the “Maternal Stress and Offspring TL” group that met our criteria of having complete beta coefficients and standard errors available for the analysis, acknowledging that statistical power to detect true moderator effects could be reduced. Despite this limited sample size, we proceeded to examine two covariates as potential moderators: (a) stress instrument type (categorized as depression scale, pregnancy specific, perceived stress, or other), and (b) measurement timing (categorized as pregnancy, not specified, or other temporal categories).

All intermediate and final output records were systematically saved for transparency and reproducibility. This included diagnostics visualizations such as forest plots, scatter plots and funnel plots of sample size against effect size, detailed logs of excluded studies with reasons for exclusion, and the results of sensitivity analysis (for e.g., checks for consistency in the direction of reported effects).

## 3. Results and Discussion

### 3.1. Baseline Characteristics of the Included Studies

In this systematic review and meta-analysis, we included 16 observational studies examining the association between maternal stress and offspring TL/DNAm age in linear regression models. All studies were published between January 2016 and June 2023. Sample sizes ranged from 24 to 1405 participants. The mean age of the mothers ranged from 24 to 39 years old. All women were aged 16 years or older with a single pregnancy. Ten studies were conducted in the United States of America (USA) [20,32,44,45,46,47,49,50,52], two in Finland, and one each in Singapore [42], Democratic Republic of Congo [31] and South Africa [33]. The outcomes of all the studies are the same: they assess how stress during pregnancy affects TL and DNAm age in the offspring (detailed recruitment information shown in Table 2). These epigenetic markers were assessed in offspring at various developmental stages ranging from birth (umbilical cord blood or neonatal saliva/blood) to early childhood (e.g., 4–18 months, 3–5 years) and even adolescence (6–16 years, depending on the study design). In the study by Esteves et al. [44], maternal stress during childhood was measured through Adverse Childhood Experience questionnaire score (ACEs). The study by Mayer et al. [45] used three scales namely: Life Events Scale (LES) to assess stress during adolescence, the Stress and Adversity Inventory for Adults (Adult STRAIN) to assess stress over the entire life-course, and a list of 14 major life stressors to assess stress occurring during the most recent pregnancy. In the study by Dye et al. [47], mothers’ total Adverse Childhood Experience questionnaire score (ACE) was used. In the study by Stout-Osvald et al. [43], pregnancy-related anxiety was assessed using the 10-item pregnancy-related anxiety scale while maternal perceived stress was measured using 10-item version of Cohen’s Perceived Stress Scale (PSS). PSS was also used by Carroll et al. [46], Send et al. [51], Verner et al. [53], Entringer et al. [20], Ämmälä et al. [48], and Izano et al. [49]. Quinn et al. [31] used the validated Early Trauma Inventory–Self Report to assess general trauma, and the Trauma History Questionnaire with the Hassles Scale to assess chronic stress. In the Koen et al. study [33], exposure to stressful events was assessed using the modified World Mental Health Life Events Questionnaire (LEQ), the Childhood Trauma Questionnaire (CTQ) and the Intimate Partner Violence (IPV) Questionnaire. Also, psychological distress was assessed using the Self-Reporting Questionnaire (SRQ-20) and the Edinburgh Postnatal Depression Scale (EPDS). The study by Katrinli et al. [32] used three different scales: LES, the Structured Trauma-Related Experiences and Symptoms Screener for Adults (STRESS-A) to assess lifetime trauma, Structured Trauma-Related Experiences and Symptoms Screener for Adults (STRESS-A), and the Difficulties in Emotion Regulation Scale (DERS) to assess emotion dysregulation. In the study conducted by Chen et al. [42], maternal stress was assessed through EPDS and STAI. Additionally, in the study conducted by Marchetto et al. [50] stress was measured using the Holmes and Rahe Stress Scale questionnaire. The Bosquet Enlow et al. [52] study assessed maternal stress through the Perceived Stress Scale, the Edinburgh Post-natal Depression scale and the 80-item Crisis in Family Systems–Revised (CRISYS-R) survey. This study analyzed the effects of maternal stress on males and females separately.

### 3.2. Results of the Studies Included in the Systematic Review

#### Relation Between Maternal Lifetime Stress and Offspring TL and DNAm

Two studies evaluated the effects of stress during childhood/adolescence on TL. In the study by Esteves et al. [44], higher maternal ACEs were associated with shorter infant TL (*p*-value = 0.021, β = −0.039, 95% CI = [−0.072, −0.006]). The study by Mayer et al. [45], which focused on maternal pregnancy stressors and newborn TL, reported a non-significant negative trend for the offspring of Caucasian mothers (*p*-value = 0.116, β = −0.158, SE = 0.01) and a non-significant, positive trend for the offspring of African American mothers (*p*-value = 0.308, β = 0.101, SE = 0.01).

In seven studies maternal stress was assessed only during pregnancy. Four of them reported a statistically significant inverse association between maternal stress and offspring biological aging. In the study by Chen et al. [42], EPDS scores showed no significant association with newborn TL (*p*-value = 0.140, β = −0.05, SE = 0.033, CI [−0.11,  0.02]). However, both STAI state score (*p*-value = 0.0293, β = −0.07, CI [−0.14,  −0.01]) and STAI trait scores (*p*-value = 0.00361, β = −0.09, SE = 0.033, CI = [−0.16, −0.03]) showed a significant negative association, indicating that higher antenatal anxiety was associated with shorter newborn TL. This inverse relation was also in the study conducted by Marchetto et al., [50] (*p*-value = 0.04, β = −0.463, SE = 0.002), by Entringer et al. [20] (*p*-value = 0.04, β = −0.516, SE = 0.047) and by Verner et al. [53] (*p*-value = 0.044, β = −0.079, SE = 0.039). Conversely, the studies by Ämmälä et al. [48] (*p*-value = 0.661, β = −0.01) and Izano et al. [49] (*p*-value = 0.29, β = −0.07, CI = [−0.15, 0.01]), showed a negative relation that was not statistically significant. Finally, the study by Bosquet Enlow et al. [52] analyzed the effects of maternal stress on males and females separately, showing that maternal stress was not significantly associated with TL in either male (β = 0.011, SE = 0.012) or in female offspring (β = −0.011, SE = 0.015) with an interaction *p*-value = 0.25.

Three studies [43,46,51] investigated the effects of maternal stress, including measures collected both during pregnancy and across the mothers’ lifetime. All three reported negative associations between prenatal maternal psychological distress and shorter offspring TL. Specifically, Stout-Oswald et al. [43] found that maternal psychological distress during pregnancy was associated with shorter child TL (*p*-value = 0.017, β = −0.333, CI = [−0.162, −0.017]). Similarly, Carroll et al. [46] observed shorter child buccal telomere length (bTL) linked to maternal psychological distress during pregnancy, with findings for both the second (*p*-value = 0.062, β = −0.258, SE = 0.02) and third trimesters (*p*-value = 0.016, β = −0.254, SE = 0.02). Send et al. [51] also identified an association between maternal psychological distress and shorter telomeres (*p*-value = 0.015, β = −0.14, SE = 0.057).

Two studies analyzed the effect of stress during pregnancy and lifetime on DNAm aging clocks. Koen et al. [33], found that maternal PTSD was significantly and inversely associated with child gestational epigenetic age (EA)-acceleration residuals at birth (*p*-value = 0.018, β = −1.95, CI [−3.57, −0.33]). Similarly, Katrinli et al. [32] reported that maternal PTSD symptoms were associated with lower gestational EA acceleration in neonates (*p*-value = 0.410, β = −0.00374, SE = 0.00172).

A further two studies evaluated the relation between stress during childhood and adolescence and DNAm aging. In the former, by Dye et al. [47], maternal stress was associated with accelerated epigenetic aging in males (*p*-value = 0.04, β = 0.06, CI [0.001, 0.11]), but not in females (*p*-value = 0.57, β = 0.01, CI [−0.04, 0.07]). The latter, by Quinn et al. [31] assessed newborn DNAm using three different clocks: Horvath’s pan-tissue, intrinsic epigenetic age, and extrinsic epigenetic age clocks. They reported that general trauma (*p*-value = 0.02, β = 0.70, SE = 0.30) and war trauma (*p*-value = 0.048, β = 1.12, SE = 0.56) were positively associated with epigenetic age acceleration only when measured by extrinsic epigenetic ageing clocks.

### 3.3. Quality Assessment Findings

The JBI tool was used to assess the risk of bias in the included studies for the systematic review and meta-analyses [54]. We used two separate JBI checklists to assess the risk of bias in cross-sectional (k = 14) and cohort studies (k = 2), respectively (Supplementary Tables S2 and S3). Overall, 12 [20,31,32,33,43,44,45,46,48,49,50,51,53] out of 16 included studies did not describe the study subjects and settings in much detail. Nevertheless, all 16 studies were overall rated as “Good” and suitable to be included in the meta-analysis (Table 2).

### 3.4. Results of the Meta-Analysis

A total of 12 studies were included in the meta-analysis categorized into six predefined exposure–outcome groups. After applying eligibility criteria and quality control procedures, meta-analyses were conducted for four groups with at least three eligible studies: (1) Maternal Perceived Stress (i.e., as mentioned above, it is often measured using a standard scale and thus implies a subjective appraisal or feeling of being stressed) [35] and offspring TL; (2) Maternal Stress and offspring TL; and (3) Maternal Stress (i.e., used as a broad term encompassing factors and emotions experienced by pregnant women) and DNAm Age Acceleration. Groups with fewer than three studies (i.e., Lifetime Stress and TL; Maternal Depression/Anxiety; Maternal PTSD and DNAm Age Acceleration; General Trauma and DNAm Age Acceleration) were excluded from the model-based synthesis.

**Figure 2:**
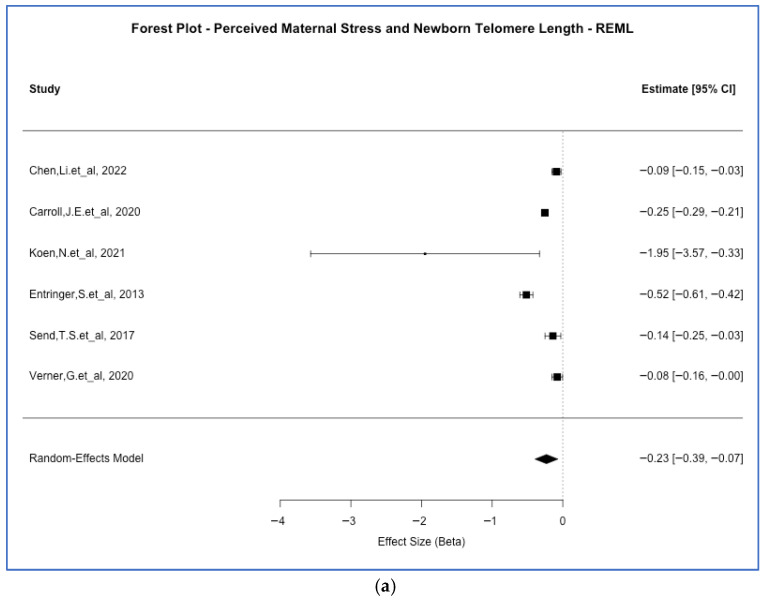

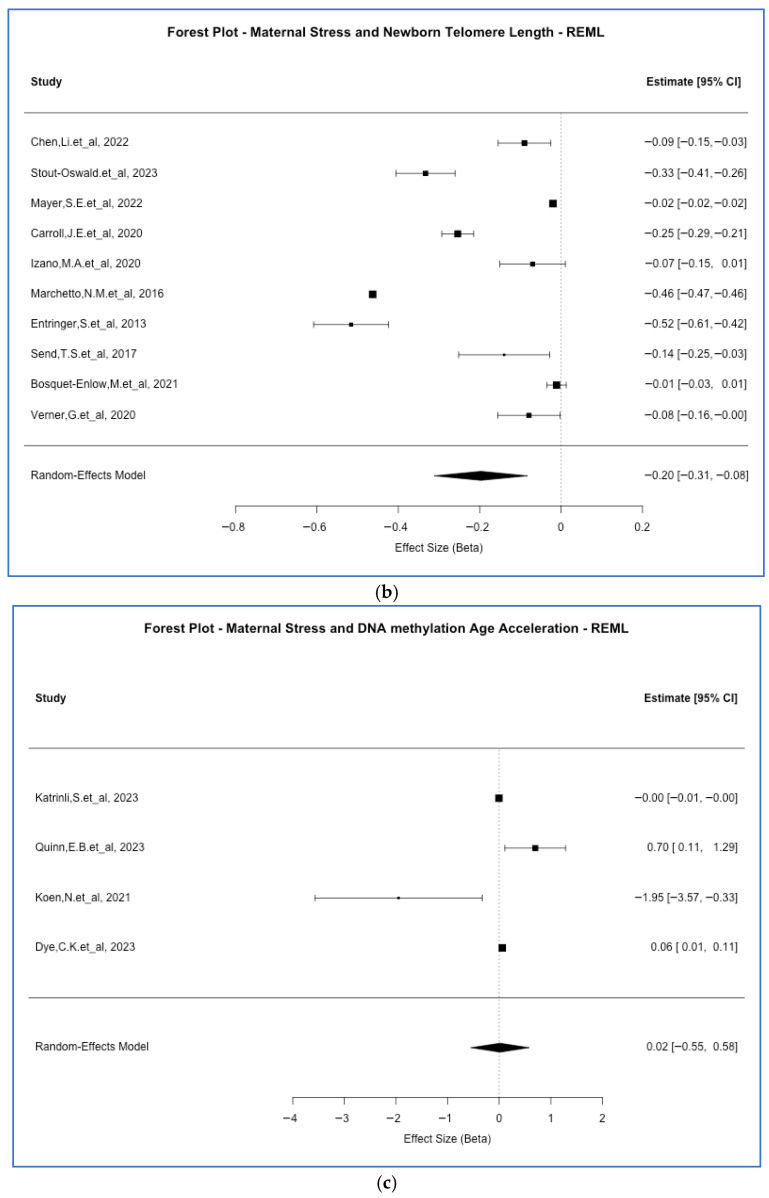
Forest plots for the different random effect meta-analyses carried out. (**a**) Perceived maternal stress and newborn telomere length Chen [42], Carroll [46], Koen [33], Entringer [20], Send [51], Verner [53]; (**b**) Maternal stress and newborn telomere length Chen [42], Stout-Oswald [43], Mayer [45], Carroll [46], Izano [49], Marchetto [50], Entringer [20], Send [51], Bosquet-Enlow [52], Verner [53]; and (**c**) Maternal stress and DNA methylation age acceleration Katrinli [32], Quinn [31], Koen [33], Dye [47].

A significant inverse association was observed between perceived maternal stress and neonatal TL. The random-effects model yielded a pooled estimate of β = −0.23 (*p*-value = 4.7 × 10^−3^, 95% CI [−0.39, −0.07]), with substantial heterogeneity (I^2^ = 95.4%). As illustrated in Figure 2a, most individual studies reported negative associations, although heterogeneity was driven in part by one large effect [33]. The sample-size weighted models produced comparable estimates, and *p*-value combination methods (Fisher’s *p*-value = 2.4 × 10^−6^; Stouffer’s *p*-value < 0.001) supported the observed association. This effect size (i.e., β = −0.23) indicates that for each SD increase in maternal perceived stress, offspring TL decreases by approximately 0.23 SD. This small to moderate effect that may have meaningful implication for long-term cellular aging trajectories, as shorter telomeres at birth have been associated with increased risk of age-related diseases later in life [64,65].

When combining all maternal stress types and TL outcomes (i.e., five studies with valid effect size data were included), we found a consistently inverse association. The REML estimate was β = −0.20 (*p*-value < 0.001, 95% CI [−0.31, −0.08]), with a very high heterogeneity (I^2^ = 99.9%). Fixed-effect and sample-size weighted models were concordant with this evidence, and Fisher’s method again supported statistical significance (*p*-value = 4.4 × 10^−6^). As seen in Figure 2b, although the effect sizes varied across studies, the majority supported a negative direction with several large magnitudes of effects which could contribute to the substantial heterogeneity. This would suggest an inverse relationship across various measures of maternal stress exposure and TL. The consistency of this inverse association (β = −0.20) across diverse maternal stress timing and exposure, including perceived stress, anxiety, depression, and trauma, suggests a common biological pathway linking maternal psychological adversity to offspring telomere attrition, irrespective of the specific stressor type. However, the very high heterogeneity (I^2^ = 99.9%) underscores substantial variability in effect magnitude across studies, likely reflecting differences in measurement approaches, population characteristics, and timing of assessments.

When the association between maternal stress and DNA methylation-based age acceleration was assessed (i.e., this was based on two studies with valid effect size data), the results were more heterogeneous. The REML model showed no significant association (*p*-value = 0.95, β = 0.02; 95% CI [−0.55, 0.58]), but the fixed-effect model indicated a marginally significant negative association (*p*-value = 0.042, β = −0.0035). While some *p*-value combination approaches (e.g., Fisher’s *p*-value = 2.9 × 10^−4^) suggested significance, the effect directions were inconsistent across studies (Figure 2c), likely contributing to the high heterogeneity (I^2^ = 81.5%) and limiting our interpretability.

Model-based meta-analysis was conducted only for groups with at least three studies containing valid beta coefficients and standard errors. Groups with insufficient data were analyzed using a *p*-value combination method only. Table 1 reports the baseline characteristics of all included studies. The corresponding statistical results and findings that served as input for the meta-analyses and subsequent analytical approaches are present in Table 4.

**Figure 3:**
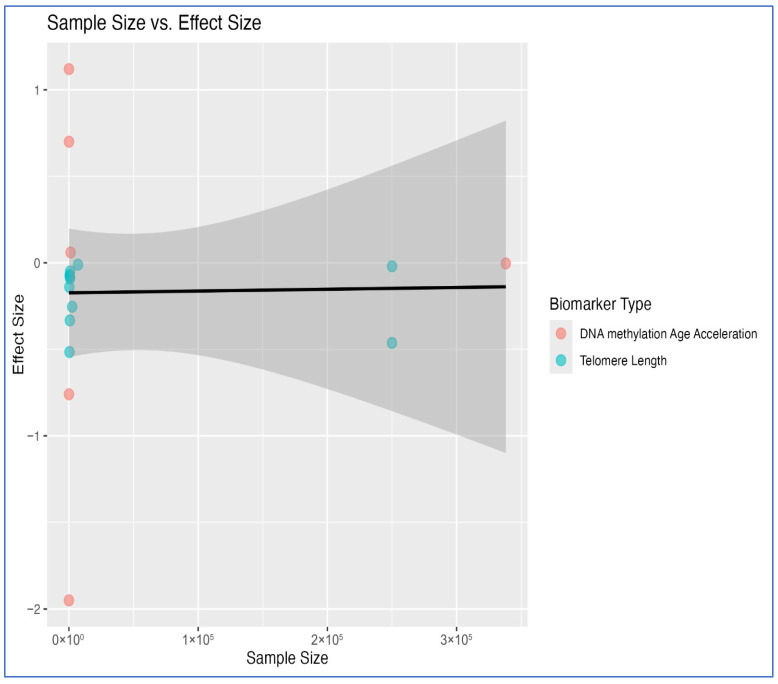
Scatter plot of sample size versus effect size. The solid line represents the regression trend. The shaded ribbon indicates the 95% confidence interval, which narrows at lower sample sizes (where data are more densely clustered) and widens at higher sample sizes (where data are sparse), reflecting greater model uncertainty in that range.

To explore potential small-study effects, a scatter plot of effect size versus sample size was generated (Figure 3). No clear pattern of funnel asymmetry was observed, though several smaller studies reported larger absolute effects, particularly in telomere-related outcomes.

**Figure 4:**
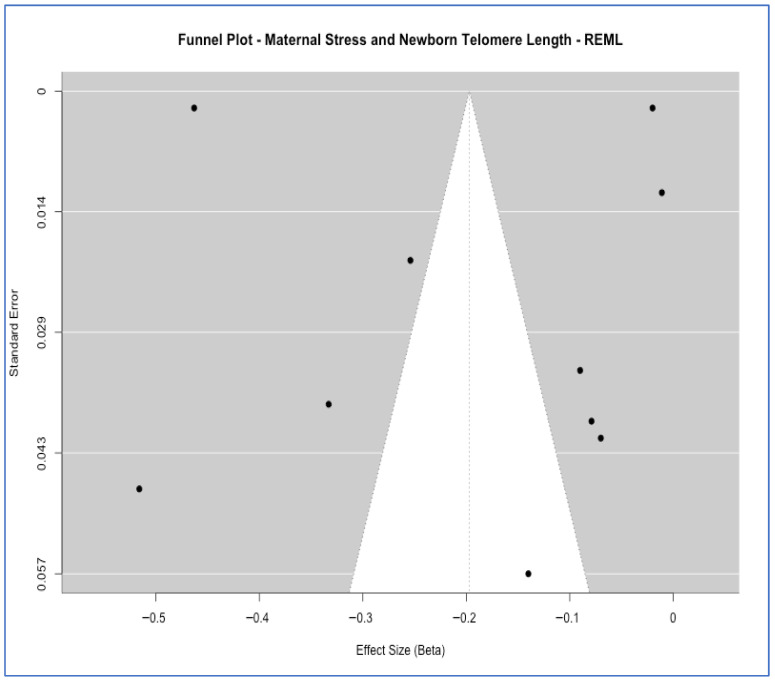
Funnel plot for the meta-analysis of the associations between maternal stress and offspring telomere length, with each point representing an individual study. The white central region represents the area of non-significance; the dark grey outer regions represent the rejection zone (*p* < 0.05). Asymmetry in the distribution of studies across these regions may indicate publication bias.

As already previously noted and illustrated in the scatter plot description (Figure 3), smaller studies on telomere-related outcomes had larger absolute effects. Visual inspection of the funnel plot for all maternal stress and offspring TL (REML model) did not reveal substantial asymmetry (Figure 4), suggesting a low risk of systematic publication bias influencing the findings for this group. Additionally, we formally tested for publication bias using Begg’s rank correlation [60,61] and Egger’s regression tests [62]. For TL studies (k = 6), Begg’s test showed no significant bias (*p*-value = 1.00) while Egger’s test suggested a potential small-study effect (*p*-value = 0.013; Supplementary Table S4). This discordance, coupled with the limited number of studies, makes definitive conclusions about publication bias challenging.

To investigate the substantial heterogeneity observed in the association between all forms of maternal stress and offspring TL, we performed a meta-regression analysis focusing on two key methodological factors namely stress instrument type and measurement timing. We tested whether these specific factors significantly influenced the effect size. Our results (k = 5) indicated that neither stress instrument type (R^2^ = 11.87%, QM [the statistical test for moderators in our model] *p*-value = 0.324) nor measurement timing (R^2^ = 0%, QM *p*-value = 0.607) explained the substantial heterogeneity we observed (I^2^ > 95%). However, we acknowledge that this analysis was limited to five studies which reduces our statistical power to detect significant moderator effects. The combined model including both covariates similarly showed no significant moderating effects (R^2^ = 11.87%, QM *p*-value = 0.324). See Supplementary Tables S1 and S5. We also searched for other potential moderators, such as offspring age at assessment, tissue source, and geographic region; however, these variables were inconsistently reported across studies or showed insufficient variability in our subset of five studies, thereby precluding a statistically meaningful analysis.

This suggests that the high heterogeneity is not explained by the type of stress assessment instrument or the timing of stress measurement, and likely arise from another, unmeasured difference.

To address possible concerns regarding the aggregation of distinct stress constructs, we performed exploratory meta-analyses for specific stressor types. For TL outcomes, analyses for perceived stress (number of studies with valid data (k = 2) and maternal stress (k = 4) both showed significant inverse associations (perceived stress: *p*-value = $6.98\times 10^{-4}$, β = −0.085; 95% CI [−0.135, −0.036]; maternal stress: *p*-value = 0.040, β = −0.136; 95% CI [−0.265, −0.006]). For DNAm age acceleration, only trauma exposure (k = 2 studies) yielded a result (*p*-value = $2.69\times 10^{-3}$, β = 0.794, 95% CI [0.275, 1.312]). Meta-analyses for TL vs. other specific constructs (e.g., lifetime stress, PTSD) were not feasible due to an insufficient number of studies (k < 3).

Forest plots for the restricted maximum likelihood (REML) meta-analyses of offspring telomere length (TL) vs. *(a)* perceived maternal stress, *(b)* all maternal stress and *(c)* DNA methylation (DNAm) age acceleration vs. all maternal stress are reported, by the two aging outcomes that were meta-analyzed. Effect size is given by association Betas between maternal stress exposures and biological aging outcomes for each analyzed study.

The plot shows the distribution of effect sizes against standard errors for included studies under a restricted maximum likelihood model (REML). The absence of substantial funnel asymmetry suggests limited evidence of small-study effects or publication bias.

### 3.5. Discussion

This systematic review and meta-analysis aimed to assess the effect of maternal stress during lifetime and pregnancy on the biological age of the offspring, assessed through DNAm age acceleration and telomere length. Our work expands upon previous systematic review [66], which focused only on prenatal stress and used only TL assessed at delivery, by including studies assessing biological aging across the offspring’s lifespan and accounting for maternal stress throughout her entire lifetime.

Our meta-analysis provides a quantitative foundation to the existing systematic literature review, reinforcing the association between a wider maternal stress exposure and multiple molecular markers of biological aging in offspring. Across the three exposure-outcome groups with sufficient data (namely, perceived stress during pregnancy and offspring TL; maternal stress and offspring TL; and maternal stress and DNAm age acceleration), we observed a consistent inverse association between several forms of maternal stress (including perceived stress, all types of stress) and TL. This pattern suggests an influence of maternal stress on telomere attrition and accelerated biological aging in the offspring, which was particularly noticeable for measures of maternal perceived stress linked to offspring TL. However, given the substantial heterogeneity in study designs, measurement approaches, and population characteristics (I^2^ > 90% in most analyses), these findings should be interpreted with caution and viewed as preliminary evidence based on a larger number of primary studies, especially between maternal stress and DNAm age clocks.

For perceived stress during pregnancy and offspring TL meta-analysis, the random-effects model yielded a significant inverse association. This finding was confirmed by a fixed-effect approach, aligning with the hypothesis that prenatal psychosocial stress may contribute to accelerated cellular aging in offspring through mechanisms involving telomere attrition [21,67].

Our meta-analysis, which aggregated studies across all maternal stress exposures and offspring TL outcomes, showed an overall negative association further emphasizing that cumulative maternal stress, regardless of its specific nature or timing, might exert lasting biological effects on offspring. This evidence not only reinforces the general association reported by Moshfeghinia and colleagues [66] for prenatal stress, but expands upon it to demonstrate that this effect extends to exposures to stress throughout a mothers’ entire life, a scope that was excluded from their prior review.

Several potential pathways may explain the identified link between prenatal exposure to maternal stress and telomere shortening potentially leading to disease onset, described elsewhere [21,66,68]. This finding aligns closely with proposed physiological pathways linking psychological stress to cellular aging. Chronic maternal stress is known to dysregulate the HPA axis, leading to elevated glucocorticoid levels. These stress hormones can cross the placenta and may directly inhibit telomerase activity, the enzyme responsible for maintaining TL [20]. Furthermore, stress-induced inflammation and oxidative stress represent another key mechanism; prenatal stress increases pro-inflammatory cytokines and reactive oxygen species (ROS), both of which can cross the placental barrier [15]. Since telomeres are highly susceptible to damage by ROS, and inflammatory signals can accelerate telomere shortening with each cell division, this provides a plausible biological pathway for our observed association [21,67]. Beyond these molecular mechanisms, an important consideration is whether the observed telomere shortening and potential epigenetic modification at birth could predispose offspring to clinical syndromes involving accelerated biological aging or other age-related disorders later in life. Although longitudinal data directly linking prenatal stress exposure to such clinical outcomes remain limited, shorter telomere length has been prospectively associated with increased risk of cardiovascular disease, type 2 diabetes, neurodegenerative disorders, and immunosenescence in adulthood [69,70,71]. Thus, the molecular signatures we observed at birth may represent early signs of elevated susceptibility to age-related pathologies across the lifespan, warranting long-term follow-up studies to test this hypothesis, in a typical DOHaD perspective.

In contrast, the findings for the meta-analysis of maternal stress and DNAm age acceleration were less clear-cut. While the random-effects model produced a non-significant result, fixed-effect models suggested a marginally significant inverse association, which did not survive correction for multiple testing, except when using *p*-value combination approaches which are not informative on the direction of the association. In contrast, the association between maternal stress and DNAm age acceleration was not statistically significant and highly heterogeneous. This divergence from the TL findings could reflect the differences in what these biomarkers capture. For instance, TL primarily reflects cumulative wear and tear caused by cellular replication [72] and chronic oxidative and inflammatory damage [67], processes that are known to be sped up by the sustained activation of physiological stress axes. In contrast, DNAm age is multi-tissue composite estimator derived from specific CpG sites across the genome, influenced by a more complex and varied set of genetic, environmental, and developmental factors [73].

The lack of a consistent signal in our DNAm analysis may indicate that the impact of maternal stress may be more highlighted in telomere dynamics than on the pan-tissue epigenetic aging clocks used in the current literature. Indeed, the discrepancy between models, coupled with high heterogeneity could likely point towards a possible methodological variability in the primary studies, such as differences in the epigenetic clocks used and the tissue sources analyzed.

An important consideration when interpreting our TL findings is the potential impact of tissue specificity on measurement and biological significance. The included studies utilize different biological sample types, including umbilical cord blood, peripheral blood, saliva, and buccal mucosa, which may not be directly comparable. Telomere attrition rates, baseline TL, and cellular composition are known to vary substantially across tissue types [74,75]. For instance, cord blood samples collected at birth primarily reflect in utero conditions and contain a high proportion of nucleated red blood cells and lymphocytes, whereas postnatal peripheral blood samples reflect both prenatal exposures and postnatal environmental influences, with different cellular distributions. Similarly, buccal mucosa and saliva samples represent epithelial tissues with distinct proliferation rates and telomere dynamics compared to blood-derived leukocytes [76]. These tissue-specific differences may contribute to the observed inconsistencies in effect sizes across studies and could explain some of the substantial heterogeneity we observed (I^2^ > 90%), although we were not able to identify a clear relationship between the tissue types analyzed and the association observed between maternal stress and TL in the primary studies meta-analyzed here (Supplementary Table S6). Furthermore, different tissue types may exhibit varying sensitivity to maternal stress exposure, with some tissues potentially showing stronger or more persistent effects than others. Future studies, where feasible, should systematically account for tissue-specific differences and examine multiple tissue types with the same cohort to better characterize the tissue-specific impact of maternal stress on offspring telomere dynamics and to determine whether effects are generalizable across biological components or tissue-restricted.

However, the extremely high heterogeneity observed underscores the significant diversity in measurement tools, populations studied, and exposure timing across the included research, as noted in previous reviews [66]. To further expand on this, we conducted a meta-regression examining two key methodological factors: stress instrument type and measurement timing. Neither factor significantly explained the heterogeneity observed in the association between maternal stress and offspring TL. This suggests that the variability in effect sizes cannot be attributed solely to differences in how stress was measured or when it was assessed during the maternal lifespan. Rather, the persistence of high heterogeneity could imply that other unmeasured sources of variability contribute to the observed variation, potentially limiting the interpretation of the pooled estimates. These may include differences in biological sample types (e.g., cord blood versus buccal cells), offspring age at assessment, population-specific characteristics, or the complex interplay between multiple stress exposures. Furthermore, methodological variation in TL assessment (qPCR vs. TRF) could also reflect the high heterogeneity, as these methods differ in their precision, measurement units, and susceptibility to technical variability [38,77]. However, a comparison of the effect sizes across the different meta-analyzed studies, the use of standardized outcome measures and of a random-effect meta-analysis approach allow us to rule out substantial modification of effect due to these factors in the analyzed relationships.

### 3.6. Strengths and Limitations

To our knowledge, this is the first systematic review and meta-analysis to account for stress not only during pregnancy but during the entire lifetime, including mothers’ childhood, adolescence, and adulthood. This wide range of measurements gives us a comprehensive idea of how the influence that stress experienced by mothers at different life stages can affect the biological age of their offspring. We kept a wide range of inclusion criteria to capture studies covering a more extended range of maternal stress exposure times and allowed for the inclusion of biological age assessments in offspring until late adolescence.

Still, the present study has some inherent shortcomings. All included studies rely on an observational design, meaning residual confounding cannot be ruled out. While most studies adjusted for major covariates (e.g., maternal age and socioeconomic status), unmeasured factors could confound the relationship between maternal stress and offspring epigenetic ageing. These could include genetic predispositions (e.g., to increased stress sensitivity and variability in epigenetic aging or telomere attrition rates), paternal influences, and the postnatal caregiving environment [28]. Furthermore, while the JBI checklist rated all studies as suitable for inclusion, the uniformly high-quality scores may not fully capture the more subtle methodological issues and/or biases inherent in observational research, a limitation of the evidence base.

A related limitation is that the included studies did not systematically screen for, or control for, underlying genetic factors, particularly germline mutations associated with premature aging syndromes. Rare genetic conditions such as dyskeratosis congenita (TERC, TERT, DKC1), Hutchinson–Gilford progeria syndrome, or Bloom syndrome, amongst others, are characterized by accelerated telomere shortening and altered DNA methylation patterns that could independently influence offspring biological ageing markers [78,79]. While such conditions are uncommon, even subclinical genetic variants affecting telomere maintenance genes or DNAm machinery could confound the observed associations [80,81]. Future studies incorporating genetic screening or family history assessments would help disentangle heritable genetic contributions from stress-related environmental effects on offspring epigenetic ageing.

The evidence base is further challenged by limitations in statistical power. Many studies, especially those on DNAm age acceleration (only two studies meta-analyzed), were conducted with a relatively small number of subjects (ranging from 24 to 1405 participants), which increases the risk that published findings could represent inflated effect sizes, thus restricting our ability to draw definitive conclusions [82]. Additionally, the heterogeneity in study context including different healthcare systems, social support structures, and stress exposures across diverse geographic settings (USA, Singapore, Finland, Germany, Democratic Republic of Congo, South Africa) could limit the extent to which findings can be extrapolated across populations. Furthermore, most included studies were cross-sectional, limiting causal inference. Similarly, the limited number of studies available for our meta-regression (k = 5) reduced our power to detect true moderator effects.

Furthermore, a conceptual limitation is our use of the broad umbrella term “maternal stress”, which combines conceptually and clinically distinct psychosocial exposures (such as perceived stress, depressive symptoms, anxiety, PTSD, and lifetime trauma). While this aggregation was a strategic choice to ensure statistical power and methodological justification for our primary analysis, it contributes to heterogeneity and may obscure unique biological pathways (e.g., HPA axis dysregulation, inflammation, or epigenetic changes) for each distinct construct. Our exploratory, stress-type-specific analysis confirmed this limitation; although some specific constructs (like perceived stress) showed individual associations with shorter offspring TL, the evidence base for more specific stressor-outcome pairs remains limited. Therefore, our primary finding should be interpreted as cautious evidence that maternal psychosocial adversity in general is associated with accelerated epigenetic aging in offspring.

Our formal publication bias assessment yielded mixed results. Egger’s test suggested potential small-study effects for the TL analysis, yet Begg’s test showed no significant bias. This contradiction, combined with the limited number of studies available for these tests, makes definitive conclusions about publication bias challenging. Caution is still warranted, as subtle reporting biases can be difficult to exclude completely, especially when studies vary widely in size and effect magnitude.

Overall, further meta-analyses with a higher number of more homogeneous studies testing DNAm age acceleration versus maternal stress are warranted to clarify this relation, especially considering the recent findings that prenatal maternal stress in rats alters DNA methylation and transcriptomic patterns across four generations [83].

Future meta-analyses would benefit from greater specificity and granularity in how maternal exposures are defined and categorized, allowing for a more nuanced understanding of their specific effects on offspring biological aging.

### 3.7. Clinical Implications and Future Research Directions

Our findings highlight several important paths for future research and potential clinical applications that extend beyond the scope of the current systematic review and meta-analysis.

A critical question for future investigation is whether the observed alterations in epigenetic markers, specifically telomere shortening and DNAm age acceleration, are reversible in life. While our study documents association between maternal stress and epigenetic markers measured at birth or early childhood, longitudinal studies tracking these biomarkers across development are needed to determine whether early interventions could normalize these molecular signatures. Emerging evidence suggests that lifestyle modifications and reduced stress exposure may slow telomere attrition in adulthood [84,85], but whether similar plasticity exists for stress-related epigenetic programming established during the prenatal period remains to be established. Understanding the potential for recovery of shortened telomere or compensation for stress-induced epigenetic changes could inform the timing and nature of interventions aimed at mitigating early-life biological aging.

The potential for prevention actions warrants careful exploration and represents a logical next step for translating our findings into clinical practice. Specific interventions during pregnancy, such as an enhanced social support system, evidence-based psychological counselling, mindfulness-based stress reduction programs, or cognitive-behavioral therapy for prenatal anxiety could potentially mitigate the intergenerational transmission of biological ageing markers [86,87]. Randomized controlled trials examining whether such interventions can prevent or slow down epigenetic aging in offspring would provide evidence for clinical practice and public health policy. Such studies would not only test the causal nature of the associations we observed but also identify possible actional strategies to promote healthier developmental trajectories for children of mothers experiencing psychological adversity. Given the growing implementation of prenatal mental health screening programs, integration of biological ageing biomarkers as outcome measures could help evaluate the long-term effectiveness of maternal psychosocial interventions.

While our meta-analysis shows associations between maternal stress and offspring biological aging markers, the underlying biological pathways required further elucidation. Future studies should investigate the specific mechanisms mediating these relationships, including the roles of maternal cortisol dysregulation, systemic inflammation, oxidative stress, placental function and nutrient transfer, and direct effects on fetal programming [5,88]. Understanding these mechanisms could identify additional intervention targets beyond stress reduction alone, e.g., anti-inflammatory approaches, antioxidant supplementation, or placental health optimization, to inform more precise, mechanism-based prevention strategies. Moreover, exploring potential sex-specific effects and identifying critical windows of vulnerability during gestation could enable more targeted interventions.

However, from a public health perspective, our findings underscore the importance of comprehensive prenatal care that addresses both physical health and maternal mental health and psychosocial wellbeing. The intergenerational transmission of stress-related biological aging has implications for reducing long-term health disparities, as maternal psychological adversity is often concentrated among socioeconomically disadvantaged populations who face structural barriers to mental health care access [89]. Integrating routine mental health screening, accessible psychological support, and stress reduction resources into standard prenatal care could represent a cost-effective strategy for promoting population health across generations. Furthermore, policies addressing the social determinants of maternal stress, including economic security, safe housing, and/or community support systems may have downstream benefits for offspring health trajectories that extend well beyond the prenatal period.

## 4. Conclusions

This study highlights the potential for a lasting intergenerational link between maternal psychosocial adversity on telomere attrition, a key molecular marker of biological aging. These findings may be of clinical importance, promoting awareness of the wide-ranging effects of stress among women and suggesting the need for more effective strategies for stress management across various life stages to mitigate potential detrimental effects on their offspring’s health.

## Figures and Tables

**Table 1:** Study characteristics.

Paper (Title)	Authors and Publication Year	Country	Sample Size	Study Design	Offspring Age at Assessment
Variability in newborn telomere length is explained by inheritance and intrauterine environment	(Chen, L. et al., 2022) [42]	Singapore	950 MT-offspring (GUSTO)	CS	Umbilical cord blood at delivery
Prenatal exposure to maternal psychological distress and telomere length in childhood	(Stout-Oswald, S.E. et al., 2022) [43]	USA	102 MT-offspring	Long-Pros	Blood spots via finger prick at the childhood lab visit
Adverse childhood experiences: implications for offspring telomere length and psychopathology.	(Esteves, K.C. et al., 2020) [44]	USA	155 MT-offspring	CS	Infant buccal swabs for DNA (4, 12, 18 months)
Intergenerational effects of maternal lifetime stressor exposure on offspring telomere length in Black and White women	(Mayer, S.E. et al., 2023) [45]	USA	155 MT-offspring (112 Black, 110 White)	Pros Cohort	Saliva
Cumulative stress, PTSD, and emotion dysregulation during pregnancy and epigenetic age acceleration in Hispanic mothers and their newborn infants	(Katrinli, S. et al., 2023) [32]	USA	89 MT-offspring	CS	Saliva (neonates, within 24 h post-delivery)
Prenatal maternal stress is associated with site-specific and age acceleration changes in maternal and newborn DNA methylation	(Quinn, E.B. et al., 2023) [31]	Democratic Republic of Congo	155 MT-offspring	CS	Venous blood from neonates
Prenatal maternal stress prospectively relates to shorter child buccal cell telomere length	(Carroll, J.E. et al., 2020) [46]	USA	111 MT-offspring	CS	Buccal cell samples (children 3–5 years old)
Maternal psychosocial risk factors and child gestational epigenetic age in a South African birth cohort study	(Koen, N. et al., 2021) [33]	Africa	271 MT-offspring	CS	Umbilical cord blood at delivery
Mother’s childhood adversity is associated with accelerated epigenetic aging in pregnancy and in male newborns	(Dye, C.K. et al., 2023) [47]	USA	785 MTs and 753 offspring (AIRES/ALSPAC)	CS	Umbilical cord blood at birth and peripheral blood at follow-up visits
Maternal stress or sleep during pregnancy are not reflected on telomere length of newborns	(Ämmälä, A.J. et al., 2020) [48]	Finland	1405 MT-offspring (CS Cohort)	CS	Blood leukocyte from the umbilical cord at birth
The association of maternal psychosocial stress with newborn telomere length	(Izano, M.A. et al., 2020) [49]	USA	355 MT-offspring (UCSF CIOB)	CS	Umbilical cord blood leukocytes at delivery
Prenatal stress and newborn telomere length	(Marchetto, N.M. et al., 2016) [50]	USA	24 MT-offspring	CS	Umbilical cord blood at delivery
Maternal psychosocial stress during pregnancy is associated with newborn leukocyte telomere length	(Entringer, S. et al., 2013) [20]	USA	27 MT-offspring	CS	Umbilical cord blood at delivery
Telomere length in newborns is related to maternal stress during pregnancy	(Send, T.S. et al., 2017) [51]	Germany	318 MT, 319 offspring	CS	Umbilical cord blood at delivery
Maternal psychosocial functioning, obstetric health history, and newborn telomere length	(Bosquet Enlow, M. et al., 2021) [52]	USA	146 MT-offspring (SPEC Study)	CS	Umbilical cord blood leukocytes at delivery
Maternal psychological resilience during pregnancy and newborn telomere length: a prospective study	(Verner, G. et al., 2021) [53]	Finland	656 MT-offspring (PREDO Cohort)	CS	Cord blood after birth

Abbreviations: MT: mother; CS: cross-sectional; Long-Pros: longitudinal-prospective; Pros Cohort: prospective cohort; GUSTO: Growing Up in Singapore Towards healthy Outcomes study; AIRES: Avon Longitudinal Study of Parents and Children stub-study; ALSPAC: Avon Longitudinal Study of Parents and Children; CS Cohort: CHILD-SLEEP cohort; UCSF CIOB: University of California, San Francisco Chemicals in Our Bodies study; SPEC Study: Spontaneous Prematurity and Epigenetics of the Cervix Study; PREDO Cohort: Prediction and Prevention of Pre-eclampsia and Intrauterine Growth Restriction cohort.

**Table 2:** Inclusion/exclusion criteria and participant details.

Paper (Title)	Authors and Publication Year	Maternal Race/Ethnicity	Offsrping Race/Ethnicity	MT Age (Avg.)	Inclusion Criteria.	Exclusion Criteria.
Variability in newborn telomere length is explained by inheritance and intrauterine environment	(Chen, L. et al., 2022) [42]	Asian (Chinese, Malays and Indians)	N/R (N/R)	30.6	Women agreed to donate birth tissues (incl. cord, placenta, cord blood) at delivery.	Not delivering in NUH/KKH, <14 wks gestation, non-homogenous parental ethnicity, not residing in Singapore 5 yrs, miscarriage, no donation intent (cord/placenta/blood), planned termination, IVF, multiple pregnancies, <18 yrs, non-Singaporean/PR, chronic disease (e.g., Type 1 DM), non-Chinese/-Malay/-Indian ethnicity
Prenatal exposure to maternal psychological distress and telomere length in childhood	(Stout-Oswald, S.E. et al., 2022) [43]	Predom. Non-Hispanic White (Mixed)	Predom. Non-Hispanic White (Mixed)	29.88	Adult, English-speaking women with singleton pregnancy	Alcohol/tobacco/drug use, neuroendocrine dysfunction, uterine/cervical/fetal anomalies
Adverse childhood experiences: implications for offspring telomere length and psychopathology.	(Esteves, K.C. et al., 2020) [44]	N/R (Mixed)	Predom. White (Mixed)	28.49	N/R	<18 yrs or non-English speakers
Intergenerational effects of maternal lifetime stressor exposure on offspring telomere length in Black and White women	(Mayer, S.E. et al., 2023) [45]	AfAm and CEU (Mixed)	AfAm and CEU (Mixed)	39	Original NGHS participant (Richmond, CA); not pregnant at recruitment; no pregnancy/miscarriage/abortion in last 3 months; not living abroad/incarcerated	N/R
Cumulative stress, PTSD, and emotion dysregulation during pregnancy and epigenetic age acceleration in Hispanic mothers and their newborn infants	(Katrinli, S. et al., 2023) [32]	Predom. White-Hispanic (Mixed)	N/R (N/R)	26.93	English-speaking, singleton pregnancy, planned delivery at specified hospital	Trauma on questionnaire, infant NICU, twins, delivered elsewhere
Prenatal maternal stress is associated with site-specific and age acceleration changes in maternal and newborn DNA methylation	(Quinn, E.B. et al., 2023) [31]	N/R (N/R)	N/R (N/R)	22.6	Singleton birth at HEAL Africa Hospital	N/R
Prenatal maternal stress prospectively relates to shorter child buccal cell telomere length	(Carroll, J.E. et al., 2020) [46]	Predom. Hispanic (Mixed)	Predom. Hispanic (Mixed)	27.1	N/R	N/R
Maternal psychosocial risk factors and child gestational epigenetic age in a South African birth cohort study	(Koen, N. et al., 2021) [33]	Black (African)	Black (African)	27	N/R	N/R
Mother’s childhood adversity is associated with accelerated epigenetic aging in pregnancy and in male newborns	(Dye, C.K. et al., 2023) [47]	AfAm, CEU, Latina (Mixed)	N/R (N/R)	29.73	N/R	N/R
Maternal stress or sleep during pregnancy are not reflected on telomere length of newborns	(Ämmälä, A.J. et al., 2020) [48]	CEU (Finns)	CEU (Finns)	30.64	Finnish-speaking families; infants born April 2011–Feb 2013; born alive at Tampere Uni. Hospital	Families outside target area
The association of maternal psychosocial stress with newborn telomere length	(Izano, M.A. et al., 2020) [49]	Predom. CEU (Mixed)	N/R (N/R)	33	English/Spanish-speaking MTs, 18–40 yrs, singleton preg., 13–27 wks gestation (2nd trim.)	N/R
Prenatal stress and newborn telomere length	(Marchetto, N.M. et al., 2016) [50]	Predom. AfAm (Mixed)	N/R (N/R)	25.1	Women 18–35 yrs; single, viable, non-anomalous infant	Pregnancy complications (hypertension, diabetes, smoking), steroid therapy, chorioamnionitis
Maternal psychosocial stress during pregnancy is associated with newborn leukocyte telomere length	(Entringer, S. et al., 2013) [20]	Predom. AfAm (Mixed)	N/R (N/R)	24	N/R	N/R
Telomere length in newborns is related to maternal stress during pregnancy	(Send, T.S. et al., 2017) [51]	CEU (Caucasian)	CEU (Caucasian)	31.5	MTs 16–45 yrs; main caregiver; German-speaking	Positive hepatitis B/C/HIV; current psychiatric disorder (inpatient); history/diagnosis of schizophrenia/psychotic disorder; substance dependency (non-nicotine) during pregnancy
Maternal psychosocial functioning, obstetric health history, and newborn telomere length	(Bosquet Enlow, M. et al., 2021) [52]	Predom. CEU (Mixed)	N/R (N/R)	32.7	≥18 yrs, <28 wks gestation, NB telomere data (cord blood), relevant psychosocial data during pregnancy, singleton gestation	N/R
Maternal psychological resilience during pregnancy and newborn telomere length: a prospective study	(Verner, G. et al., 2021) [53]	CEU (Finns)	CEU (Finns)	33.24	Singleton, intrauterine pregnancy; 1st ultrasound screening at 12 + 0–13 + 6 wks gestation	Asthma (diagnosed), ASA allergy, tobacco smoking, previous peptic ulcer/placental ablation, IBD (Crohn’s/UC), rheumatoid arthritis, hemophilia/thrombophilia, gestational weeks < 12 + 0 or >14 + 0, multiple pregnancy

Abbreviations: N/R: Not reported; preg.: pregnancy; wks: weeks; AfAm: African American; CEU: Utah Residents (CEPH) with Northern and Western European Ancestry; NUH: National University Hospital; KKH: Kandang Kerbau Hospital; NGHS: National Heart, Lung, and Blood Institute Growth and Health Study; MT: Mothers; NICU: neonatal intensive care unit.

**Table 3:** Maternal stress exposure and measurement.

Paper (Title)	Authors and Publication Year	Sample Type	MT Stress Exposure	Exposure Time	Measurement of MT Stress	Measurement Time	Stat. Model
Variability in newborn telomere length is explained by inheritance and intrauterine environment	(Chen, L. et al., 2022) [42]	Umbilical cord blood at delivery	MT perceived stress	during pregnancy	EPDS (10-items) for depressive symptoms; STAI (40-items) for anxiety	26–28 weeks	Lin. Reg.
Prenatal exposure to maternal psychological distress and telomere length in childhood	(Stout-Oswald, S.E. et al., 2022) [43]	Blood spots via finger prick at the childhood lab visit	MT perceived stress	during pregnancy and lifetime (postpartum)	Pregnancy-related anxiety scale (10-item); CES-D (9-item) for depressive symptoms; PSS (10-item) for perceived stress	5 times during pregnancy, 12 weeks post-partum, at child TL measurement (6–16 yrs)	Mult. Lin. Reg.
Adverse childhood experiences: implications for offspring telomere length and psychopathology	(Esteves, K.C. et al., 2020) [44]	Infant buccal swabs for DNA (4, 12, 18 months)	MT perceived stress	during childhood	MT ACEs using ACE questionnaire score; Prenatal MT stress using Pregnancy-Related Anxiety Scale, Chronic Strain Questionnaire, Prenatal Life Events Scale–Revised, PSS (4-item), prenatal depression (EPDS 10-item); MT depression (18 months) using BDI-II	Prenatal assessment, at 4, 12, 18 months	Lin. Reg.
Intergenerational effects of maternal lifetime stressor exposure on offspring telomere length in Black and White women	(Mayer, S.E. et al., 2023) [45]	Saliva	MT perceived stress	during adolescence, pregnancy, and across the lifespan	Adolescent stressors: LES; pregnancy stressors: list of 14 major life stressors (CDC); Lifetime stressors: Adult STRAIN (midlife)	Age 17/18 and 19/20 (adolescence), midlife (MT age = 39)	Lin. Reg.
Cumulative stress, PTSD, and emotion dysregulation during pregnancy and epigenetic age acceleration in Hispanic mothers and their newborn infants	(Katrinli, S. et al., 2023) [32]	Saliva (neonates, within 24 h post-delivery)	MT perceived stress	during past year and lifetime	Past-year stressful life events: Turner LES; lifetime trauma/DSM-5 PTSD symptoms: STRESS-A; emotion dysregulation: DERS	Third trimester	Lin. Reg.
Prenatal maternal stress is associated with site-specific and age acceleration changes in maternal and newborn DNA methylation	(Quinn, E.B. et al., 2023) [31]	Venous blood from neonates	General trauma, sexual trauma, war trauma, chronic stress	from childhood to present	General trauma: ETI-SR; chronic stress: Trauma History Questionnaire, Hassles Scale	Within one day of delivery	Mult. Lin. Reg.
Prenatal maternal stress prospectively relates to shorter child buccal cell telomere length	(Carroll, J.E. et al., 2020) [46]	Buccal cell samples (children 3–5 years old)	MT perceived stress	pre-pregnancy, during pregnancy, post-pregnancy	PSS (10-items)	3x prior to conception, 2nd trimester, 3rd trimester, 1-month post-partum	Lin. Reg.
Maternal psychosocial risk factors and child gestational epigenetic age in a South African birth cohort study	(Koen, N. et al., 2021) [33]	Umbilical cord blood at delivery	Trauma/stressor exposure; PTSD; depression; psychological distress; alcohol/tobacco use	during pregnancy and lifetime	Stressful events: modified World Mental Health LEQ, CTQ, IPV Questionnaire; psychological distress: SRQ-20; depression: BDI-II, EPDS	28–32 weeks’ gestation	Lin. Reg.
Mother’s childhood adversity is associated with accelerated epigenetic aging in pregnancy and in male newborns	(Dye, C.K. et al., 2023) [47]	Umbilical cord blood at birth and peripheral blood at follow-up visits	MT adverse childhood experiences (ACE)	during childhood	Retrospective self-reports of MT ACEs	18–32 weeks’ gestation; offspring approx. 3 years old	Lin. Reg
Maternal stress or sleep during pregnancy are not reflected on telomere length of newborns	(Ämmälä, A.J. et al., 2020) [48]	Blood leukocyte from the umbilical cord at birth	MT perceived stress, sleep quality, depression, anxiety	during pregnancy	MT stress: PSS (4-item); MT depression: CES-D; MT anxiety: STAI (Short Trait); Sleep quality: BNSQ	Third trimester (gestation week 32)	Lin. Reg.
The association of maternal psychosocial stress with newborn telomere length	(Izano, M.A. et al., 2020) [49]	Umbilical cord blood leukocytes at delivery	MT perceived stress	during pregnancy	MT stress: PSS-4	Second trimester of pregnancy	Lin. Reg.
Prenatal stress and newborn telomere length	(Marchetto, N.M. et al., 2016) [50]	Umbilical cord blood at delivery	MT perceived stress	during pregnancy	MT psychosocial stress: HRSS (43-items)	At delivery	Lin. Reg.
Maternal psychosocial stress during pregnancy is associated with newborn leukocyte telomere length	(Entringer, S. et al., 2013) [20]	Umbilical cord blood at delivery	MT perceived stress	during pregnancy	Pregnancy-specific stress scale (10 items)	Avg. 9.2 weeks gestation	Lin. Reg.
Telomere length in newborns is related to maternal stress during pregnancy	(Send, T.S. et al., 2017) [51]	Umbilical cord blood at delivery	MT perceived stress	during pregnancy and lifetime	MT stress: PSS (14 item)	Three measurement time-points: end of 3rd trimester, immediately post-delivery, 6 months post-delivery	Lin. Reg.
Maternal psychosocial functioning, obstetric health history, and newborn telomere length	(Bosquet Enlow, M. et al., 2021) [52]	Umbilical cord blood leukocytes at delivery	MT perceived stress	during pregnancy	MT stressor exposures: CRISYS-R (80-item); MT stress: PSS (4 item); Pregnancy concerns: PRAS (7 item); MT depressive symptoms: EPDS (10 item); MT psychological resilience: CD-RISC (25 item); MT general anxiety: STAI (10 item trait)	2nd trimester (77%), 3rd trimester (16%), 1st trimester (6%)	Lin. Reg.
Maternal psychological resilience during pregnancy and newborn telomere length: a prospective study	(Verner, G. et al., 2021) [53]	Cord blood after birth	MT perceived stress	during pregnancy	MT stress: PSS, EPDS, CRISYS-R (80 item)	Throughout pregnancy	Lin. Reg.

Abbreviations: TL: telomere length; MT: maternal; EPDS: Edinburgh Postnatal Depression Scale: STAI: Spielberger State-Trait Anxiety Inventory; CES-D: Centre for Epidemiological Studies-Depression Inventory; PSS: Perceived Stress Scale; ACEs: Adverse Childhood Experiences; LES: Life Events Scale; Adult STRAIN: Stress and Adversity Inventory for Adults; PTSD: Post-Traumatic Stress Disorder; STRESS-A: Structured Trauma-Related Experiences and Symptoms Screener for Adults; DERS: Difficulties in Emotion Regulation Scale; ETI-SR: Early Trauma Inventory-Self Report; CTQ: Childhood Trauma Questionnaire; IPV: intimate partner violence; LEQ: World Mental Health Life Events Questionnaire; SRQ-20: Self-Reporting Questionnaire-20; BDI-II: Beck Depression Inventory-II; BNSQ: Basic Nordic Sleep Questionnaire; HRSS: Holmes and Rahe Stress Scale (Social Readjustment Rating Scale); PSS-4: Perceived Stress Scale (4-item); CRISYS-R: Crisis in Family Systems–Revised; PRAS: Pregnancy-Related Anxiety Scale; CD-RISC: Connor–Davidson Resilience Scale; Lin. Reg.: linear regression; Mult. Lin. Reg.: multiple linear regression.

**Table 4:** Outcomes and main findings.

Paper (Title)	Authors and Publication Year	Outcome	Covariates	Main Results	Key Conclusion
Variability in newborn telomere length is explained by inheritance and intrauterine environment	(Chen, L. et al., 2022) [42]	TL	Sex, ethnicity	EPDS scores (*p*-value = 0.140, β = −0.05, SE = 0.033, CI = [−0.11, 0.02]). STAI state scores (*p*-value = 0.0293, β = −0.07, CI = [−0.14, −0.01]). STAI trait scores (*p*-value = 0.00361, β = −0.09, SE = 0.033, CI = [−0.16, −0.03]).	EPDS no assoc. with NB TL; STAI scores strong neg. corr. with NB TL, higher antenatal anxiety assoc. with shorter NB TL
Prenatal exposure to maternal psychological distress and telomere length in childhood	(Stout-Oswald, S.E. et al., 2022) [43]	TL	Child sex, child age, child BMI percentile, child life events, MT age at delivery, MT gestational weight gain, parity, gestational age at birth, MT distress post-partum and at TL assessment	MT stress during pregnancy and TL (*p*-value = 0.017, β = −0.333, t (102) = −2.44, CI = [−0.162, −0.017]). MT stress post-partum (*p*-value = 0.191, β = 0.211, CI = [−0.026, −0.121]). MT distress at time of TL assessment and TL (*p*-value = 0.715, β = 0.045, CI = [−0.046, 0.067])	MT psychological distress during pregnancy predicted shorter child TL (adj. for covariates)
Adverse childhood experiences: implications for offspring telomere length and psychopathology	(Esteves, K.C. et al., 2020) [44]	TL	TL	MT ACE (*p*-value = 0.021. β = –0.039, CI = [−0.072, –0.006])	Higher MT ACEs associated with shorter infant TL
Intergenerational effects of maternal lifetime stressor exposure on offspring telomere length in Black and White women	(Mayer, S.E. et al., 2023) [45]	TL	Child age, gender, MT TL	MT pregnancy stressors and NB TL for White MTs (*p*-value = 0.116, β = −0.158, SE = 0.01) and for Black MTs (*p*-value = 0.308, β = 0.101, SE = 0.01)	MT pregnancy stressors and race on offspring TL showed neg. assoc. for White MTs’ offspring (not significant); adolescent stressors did not interact with MT race for offspring TL
Cumulative stress, PTSD, and emotion dysregulation during pregnancy and epigenetic age acceleration in Hispanic mothers and their newborn infants	(Katrinli, S. et al., 2023) [32]	DNAm (Haftorn clock)	Proportions of monocyte, B cell, NK, CD4+T, epithelial cells	MT PTSD symptoms and EA acceleration in neonates (gestational EA acceleration: *p*-value = 0.410, β = −0.00374, SE = 0.00172)	MT PTSD symptoms associated with lower gestational EA acceleration in neonates
Prenatal maternal stress is associated with site-specific and age acceleration changes in maternal and newborn DNA methylation	(Quinn, E.B. et al., 2023) [31]	DNAm (Horvath’s pan-tissue clock, Intrinsic DNAm age, Extrinsic DNAm age)	MT BMI, MT age, parity, delivery mode, alcohol use in pregnancy, recruitment site, gestational age, 1st 2 PCs of cell type variation	General trauma (*p*-value = 0.02, β = 0.70, SE = 0.30) and war trauma (*p*-value = 0.048, β = 1.12, SE = 0.56) and EA acceleration	General trauma and war trauma positively associated with extrinsic EA acceleration
Prenatal maternal stress prospectively relates to shorter child buccal cell telomere length	(Carroll, J.E. et al., 2020) [46]	TL	Child age, batch, MT pre-pregnancy BMI	MT perceived stress: 2nd trimester (*p*-value = 0.062, β = −0.258, SE = 0.02); 3rd trimester (*p*-value = 0.016, β = −0.254, SE = 0.02); postpartum (*p*-value = 0.36, β = −0.91, SE = 0.02); preconception (*p*-value = 0.126, β = −1.56, SE = 0.02)	MT stress in late pregnancy prospectively assoc. with reduced child buccal TL at 3–5 yrs, independent of postpartum/concurrent stress
Maternal psychosocial risk factors and child gestational epigenetic age in a South African birth cohort study	(Koen, N. et al., 2021) [33]	DNAm	Study site, child sex, head circumference at birth, birthweight, mode of delivery, MT estimated household income, BMI at enrolment, HIV status, anemia, psychological distress, prenatal tobacco/alcohol use	MT PTSD and offspring gestational EA (*p*-value = 0.018, β = −1.95, CI = [−3.57, −0.33])	MT PTSD significantly and negatively assoc. with offspring gestational EA residuals at birth
Mother’s childhood adversity is associated with accelerated epigenetic aging in pregnancy and in male newborns	(Dye, C.K. et al., 2023) [47]	DNAm	Sample type, gestational age at birth, MT age, parity, smoking status (during pregnancy), education, pre-pregnancy BMI	MT stress assoc. with accelerated EA in males (*p*-value = 0.04, β = 0.06, CI = [0.001, 0.110]), not in females (*p*-value = 0.57, β = 0.01, CI = [−0.04, 0.07])	MT total ACE score assoc. with accelerated EA (Bohlin clock) in males, not females
Maternal stress or sleep during pregnancy are not reflected on telomere length of newborns	(Ämmälä, A.J. et al., 2020) [48]	TL	MT anxiety, depression, stress, BMI, Sleep, qPCR analysis plate, MT smoking, offspring’s gestational age at birth, offspring’s gender	MT perceived stress during pregnancy (PSS) (*p*-value = 0.661, β = −0.01, SE = 0.00)	Impossible to replicate findings related to PSS
The association of maternal psychosocial stress with newborn telomere length	(Izano, M.A. et al., 2020) [49]	TL	MT age, education, parity, race/ethnicity, hospital of delivery	MT perceived stress during pregnancy (PSS) (*p*-value = 0.29, β = −0.07, CI = [−0.15, 0.01])	Perceived stress marginally assoc. with shorter offspring TL, but not significant after adj. for multiple comparisons
Prenatal stress and newborn telomere length	(Marchetto, N.M. et al., 2016) [50]	TL	MT age, gestational age at birth, birthweight	Significant neg. assoc. between MT stress and offspring TL (*p*-value = 0.04, β = −0.463, SE = 0.002)	Higher MT stress levels assoc. with shorter average cord blood TL in offspring
Maternal psychosocial stress during pregnancy is associated with newborn leukocyte telomere length	(Entringer, S. et al., 2013) [20]	TL	Gestational age at birth, weight, sex, exposure to antepartum obstetric complications	Neg. relationship between pregnancy-specific stress and offspring TL (*p*-value = 0.04, β = −0.516, SE = 0.047)	Significant, independent, linear effect of pregnancy-specific stress on offspring LTL
Telomere length in newborns is related to maternal stress during pregnancy	(Send, T.S. et al., 2017) [51]	TL	Batch effects, DNA extraction method, MT age	MT perceived stress during pregnancy (PSS) (*p*-value = 0.015, β = − 0.14, SE = 0.057); MT lifetime PD (self-report) (*p*-value = 0.055, β = − 0.11)	MT stress during pregnancy (PSS) but not MT lifetime PD assoc. with shorter telomeres in offspring
Maternal psychosocial functioning, obstetric health history, and newborn telomere length	(Bosquet Enlow, M. et al., 2021) [52]	TL	MT history of smoking, pre-eclampsia in prior pregnancy, timing of MT questionnaire completion	Stress telomeres in male offspring (β = 0.011, SE = 0.015) and in females (β = −0.011, SE = 0.012) with interaction *p*-value = 0.25.	Elevated MT stress/mental health symptoms predicted longer offspring TL among males. Assoc. not significant
Maternal psychological resilience during pregnancy and newborn telomere length: a prospective study	(Verner, G. et al., 2021) [53]	TL	MT perceived stress measured using PSS (10-item)	MT stress (*p*-value = 0.044, β = −0.079, SE = 0.039)	MT stress significantly predicted shorter NB TL

Abbreviations: TL: telomere length; DNAm: DNA methylation; MT: maternal; β: beta; SE: standard error.

## Data Availability

The R scripts used for meta-analysis and meta-regression are made available in our GitHub (link https://github.com/lorettomunozvenegas/Impact-of-maternal-lifetime-stress-on-offspring-biological-aging, accessed on 1 March 2026).

## Supplementary Materials

The following supporting information can be downloaded at: https://www.mdpi.com/article/10.3390/ijms27073019/s1.
