# Supplementary material for: Impact of Maternal Lifetime Stress on Offspring Biological Aging: A Systematic Review and Meta-Analysis of Observational Studies
Source: Int J Mol Sci. 2026 Mar 26;27(7):3019. doi: 10.3390/ijms27073019 (PMC13072815; doi:10.3390/ijms27073019)
Supplement: Supplementary file 1 [file ijms-27-03019-s001.zip › ijms-4115371-supplementary.pdf]

Supplementary Materials

Supplementary Table S1: Systematic search strategy (30 November 2024)

**Database**	**Search Strategy**
**EMBASE (via Ovid)**<br>*Includes MEDLINE*	1. exp Mothers/ OR exp Pregnant Women/ OR (maternal OR pregnancy OR prenatal OR antenatal).ti,ab,kw.<br>2. exp Stress, Psychological/ OR exp Depression/ OR exp Anxiety/ OR (stress OR "psychological distress" OR adversity OR trauma OR depression OR anxiety).ti,ab,kw.<br>3. exp Telomere/ OR exp DNA Methylation/ OR "Epigenetic Clock"/ OR ("telomere length" OR telomere* OR "DNA methylation" OR "epigenetic clock" OR "biological age" OR "biological aging").ti,ab,kw.<br>4. exp Child/ OR exp Infant, Newborn/ OR (child* OR newborn OR infant* OR offspring).ti,ab,kw.<br>5. 1 AND 2 AND 3 AND 4<br>6. limit 5 to human
**Scopus**	TITLE-ABS-KEY ((maternal OR pregnancy OR prenatal OR antenatal) AND (stress OR "psychological distress" OR adversity OR trauma OR depression OR anxiety) AND ("telomere length" OR telomere OR "dna methylation" OR "epigenetic clock" OR "biological age" OR "biological aging") AND (child* OR newborn OR infant* OR offspring)) AND (LIMIT-TO (DOCTYPE , "ar"))
**PubMed**	(("Mothers"[Mesh] OR "Pregnant Women"[Mesh] OR maternal[tiab] OR pregnancy[tiab] OR prenatal[tiab] OR antenatal[tiab]) AND ("Stress, Psychological"[Mesh] OR "Depression"[Mesh] OR "Anxiety"[Mesh] OR stress[tiab] OR "psychological distress"[tiab] OR adversity[tiab] OR trauma[tiab] OR depression[tiab] OR anxiety[tiab]) AND ("Telomere"[Mesh] OR "DNA Methylation"[Mesh] OR "Telomere Homeostasis"[Mesh] OR "telomere length"[tiab] OR telomere*[tiab] OR "DNA methylation"[tiab] OR "epigenetic clock"[tiab] OR "biological age"[tiab] OR "biological aging"[tiab]) AND ("Child"[Mesh] OR "Infant, Newborn"[Mesh] OR child*[tiab] OR newborn[tiab] OR infant*[tiab] OR offspring[tiab])) NOT (editorial[pt] OR letter[pt] OR review[pt])

Supplementary Table S1 Footnotes:

Databases: EMBASE (via Ovid), MEDLINE (via Ovid), PubMed, Scopus

Search Date: November 30, 2024

Filters: Human studies; no date or language restrictions

Supplementary Table S2: Analysis of the JBI quality criteria for cross-sectional studies

**Criteria**	Chen et al., 2022	Esteves et al., 2020	Katrinl et al., 2023	Quinn et al., 2023	Carrol et al., 2020	Koen et al., 2021	Dye et al., 2023	Ämmälä et al., 2020	Izano et al., 2020	Marchetto et al., 2016	Entringer et al.,2013	Send et al., 2017	Enlow et al., 2021	Verner et al., 2021
Were the criteria for inclusion in the sample clearly defined?	Y	Y	Y	Y	Y	Y	Y	Y	Y	Y	Y	Y	Y	Y
Were the study subjects and the setting described in detail?	Y	Y	Y	Y	Y	Y	Y	Y	Y	Y	Y	Y	Y	Y
Was the exposure measured in a valid and reliable way?	Y	Y	Y	Y	Y	Y	Y	Y	Y	Y	Y	Y	Y	Y
Were objective, standard criteria used for measurement of the condition?	Y	Y	Y	Y	Y	Y	Y	Y	Y	Y	Y	Y	Y	Y
Were confounding factors identified?	Y	Y	Y	Y	Y	Y	Y	Y	Y	Y	Y	Y	Y	Y
Were strategies to deal with confounding factors stated?	Y	Y	Y	Y	Y	Y	Y	Y	Y	Y	Y	Y	Y	Y
Were the outcomes measured in a valid and reliable way?	Y	Y	Y	Y	Y	Y	Y	Y	Y	Y	Y	Y	Y	Y
Was appropriate statistical analysis used?	Y	Y	Y	Y	Y	Y	Y	Y	Y	Y	Y	Y	Y	Y

Supplementary Table S3: Analysis of the JBI quality criteria for cohort studies

**Criteria**	Stout-Osvald et al.,2022	Mayer et al., 2022
Were the two groups similar and recruited from the same population?	Y	Y
Were the exposures measured similarly to assign people to both exposed and unexposed groups?	Y	Y
Was the exposure measured in a valid and reliable way?	Y	Y
Were confounding factors identified?	Y	Y
Were strategies to deal with confounding factors stated?	Y	Y
Were the groups/participants free of the outcome at the start of the study (or at the moment of exposure)?	Y	Y
Were the outcomes measured in a valid and reliable way?	Y	Y
Was the follow up time reported and sufficient to be long enough for outcomes to occur?	Y	Y
Was follow up complete, and if not, were the reasons to loss to follow up described and explored?	Y	Y
Were strategies to address incomplete follow up utilized?	NA	NA
Was appropriate statistical analysis used?	Y	Y

Supplementary Table S4: Publication Bias Assessment for Maternal Stress and Offspring Epigenetic Aging

Group	Number of studies (k)	Begg's Test (p-value)	Egger's Test (p-value)	Trim-and-Fill (k missing)	Fail-safe N	Interpretation
TL studies	6	1.00	0.013	0	22,814	Inconclusive: Discordant results between tests; limited statistical power.
DNAm studies	3	1.00	0.005	NA	NA	Underpowered: Insufficient studies for reliable assessment.

Abbreviations: **TL**: Telomere Length; **DNAm studies**: DNA methylation age acceleration

Supplementary Table S4 Footnotes:

Begg's Test: Rank correlation test (Kendall's tau) assessing funnel plot asymmetry; p-value < 0.05 suggests potential publication bias.

Egger's Test: Linear regression test of funnel plot asymmetry; p-value < 0.05 suggests potential small-study effects.

Trim-and-Fill: A method to estimate and adjust for missing studies in meta-analysis; "k missing" indicates number of theoretically missing studies needed for symmetry.

Fail-safe N: Is the number of null studies required to overturn significant findings; larger values indicate greater strength against publication bias.

NA: Not applicable, meaning insufficient studies (k < 4) for reliable trim-and-fill analysis and fail-safe N calculation.

Supplementary Table S5: Meta-regression Summary Table

Moderator	Category	Number of studies	Coefficient (ß)	SE	p-value	R^2 (%)	QM (df) p-value	Conclusion
Stress instrument type		5				11.87	3.47 (3) p-value = 0.324	No significant moderator
	Intercept (Reference: Spielberger STAI)	-0.050	0.179	0.780				
	Other	-0.413	0.250	0.099				
	Perceived Stress	-0.020	0.254	0.937				
	Pregnancy-Specific	-0.156	0.219	0.476				
Measurement timing		5				0.00	0.26 (1) p-value = 0.607	No significant moderator
	Intercept (Reference: postpartum)		-0.259	0.147	0.078			
	During pregnancy		0.098	0.191	0.607			
Combined model (Instrument & Timing)		5				11.87	3.47 (3) p-value = 0.324	No significant moderating effect. The model did not explain a substantial amount of heterogeneity.

Abbreviations: **β**: beta, i.e. the regression coefficient (estimates the change in effect size per unit change in the moderator); **SE**, standard error; **R^2**: is the proportion of between-study variance (heterogeneity) explained by the moderator(s); **QM**, Cochran's Q-statistic for moderators (test of model significance); **df**: degrees of freedom.

Supplementary Table S6: Telomere Length Measurement Methods and Effect Sizes for Maternal Stress Associations in Meta-Analysed Studies

Reference	Sample	Age	TL assay	Beta
Bosquet-Enlow,M.et_al, 2021	umbilical cord leukocyte	newborn	qPCR	-0,01
Carroll,J.E.et_al, 2020	buccal cell	3-5 years	qPCR	-0,25
Entringer,S.et_al, 2013	PBMCs from cord blood	newborn	qPCR	-0,52
Izano,M.A.et_al, 2016	umbilical cord leukocytes	newborn	qPCR	-0,07
Marchetto,N.M.et_al, 2016	umbilical cord blood	newborn	TRF	-0,46
Mayer,S.E.et_al, 2022	buccal cell	2-17 years	qPCR	-0,02
Stout-Oswald.et_al, 2023	blood spots via finger prick	6-16 years	qPCR	-0,33
Verner,G.et_al, 2020	leukocytes from cord blood	newborn	qPCR	-0,08
Send,T.S.et_al, 2017	umbilical cord blood	newborn	qPCR	-0,14
Chen,Li.et_al, 2022	umbilical cord tissue	newborn	qPCR	-0,09

Abbreviations: qPCR = quantitative real-time polymerase chain reaction; TRF = Telomere Restriction Fragment; PBMCs = Human peripheral blood mononuclear cells.

7.1. Author Contributions

AG, SS and LI conceived and supervised the study; MSQ and SS carried out literature search; MLMV carried out the statistical analyses; MSQ, SS and AG assessed the data extracted and selected the studies for inclusion after independent assessment, and mutual agreement as applicable, and conducted the quality assessment of the studies; MSQ, MLMV, SS and AG wrote a first draft of the manuscript; PDD, FB, FG, CC, MBD, GdG. and LI critically reviewed the manuscript. All authors have read and agreed to the published version of the manuscript.

7.2.Funding

This work was partially supported by the project "Influence of Mediterranean Dietary Habits on Pregnancy and Foetus Development: the role of epigenetics and inflammation—MED-BORN", funded by the Italian Ministry of Education - Progetti di Ricerca di Rilevante Interesse Nazionale (PRIN) Bando 2022—grant 2022MMPRT3. AG was funded by the PON "Ricerca e Innovazione" 2014–2020 Programme of the Italian Ministry of Education and Research (D.M. 1062/2021), based on ESF REACT EU funds and by the Italian Ministry of Health (Ricerca Finalizzata U40, project GR-2021-12375341).

7.3.Financial disclosure

All the authors declare that there are no conflicts of interest and that the funders were not involved or had restrictions regarding the publication.

7.4.Data Availability Statement: Data availability statement

The R scripts used for meta-analysis and meta-regression are made available in our GitHub (link <https://github.com/lorettomunozvenegas/Impact-of-maternal-lifetime-stress-on-offspring-biological-aging>).

7.5.Acknowledgments

We are grateful to the participants to the Neuromed Clinical Research Network in the framework of the Big Data and Personalized Health Project.

SS and MLMV were supported by the Joint Platform Umberto Veronesi Foundation-Research Unit of Epidemiology and Prevention at IRCCS Neuromed in Pozzilli, Italy.

7.6.Conflicts of Interest

"The authors declare no conflicts of interest" and "The funders had no role in the design of the study; in the collection, analyses, or interpretation of data; in the writing of the manuscript; or in the decision to publish the results".
